# Differentiating Psychopathy from General Antisociality Using the P3 as a Psychophysiological Correlate of Attentional Allocation

**DOI:** 10.1371/journal.pone.0050339

**Published:** 2012-11-16

**Authors:** Inti A. Brazil, Robbert Jan Verkes, Bart H. J. Brouns, Jan K. Buitelaar, Berend H. Bulten, Ellen R. A. de Bruijn

**Affiliations:** 1 Donders Institute for Brain, Cognition and Behaviour, Radboud University, Nijmegen, The Netherlands; 2 Pompestichting, Nijmegen, The Netherlands; 3 Radboud University Nijmegen Medical Centre, Donders Institute for Brain, Cognition and Behaviour, Department of Psychiatry, Nijmegen, The Netherlands; 4 Department of Pedagogical and Educational sciences, University of Utrecht, Utrecht, The Netherlands; 5 Radboud University Nijmegen Medical Centre, Donders Institute for Brain, Cognition and Behaviour, Department of Cognitive Neuroscience, Nijmegen, The Netherlands; 6 Leiden University, Department of Clinical, Health and Neuropsychology, Leiden Institute for Brain and Cognition, Leiden, The Netherlands; Institute of Psychiatry at the Federal University of Rio de Janeiro, Brazil

## Abstract

Recent studies have shown that while psychopathy and non-psychopathic antisociality overlap, they differ in the extent to which cognitive impairments are present. Specifically, psychopathy has been related to abnormal allocation of attention, a function that is traditionally believed to be indexed by event-related potentials (ERPs) of the P3-family. Previous research examining psychophysiological correlates of attention in psychopathic individuals has mainly focused on the parietally distributed P3b component to rare targets. In contrast, very little is known about the frontocentral P3a to infrequent novel events in psychopathy. Thus, findings on the P3 components in psychopathy are inconclusive, while results in non-psychopathic antisocial populations are clearer and point toward an inverse relationship between antisociality and P3 amplitudes. The present study adds to extant literature on the P3a and P3b in psychopathy by investigating component amplitudes in psychopathic offenders (N = 20), matched non-psychopathic offenders (N = 23) and healthy controls (N = 16). Also, it was assessed how well each offender group was able to differentially process rare novel and target events. The offender groups showed general amplitude reductions compared to healthy controls, but did not differ mutually on overall P3a/P3b amplitudes. However, the psychopathic group still exhibited normal neurophysiological differentiation when allocating attention to rare novel and target events, unlike the non-psychopathic sample. The results highlight differences between psychopathic and non-psychopathic offenders regarding the integrity of the neurocognitive processes driving attentional allocation, as well as the usefulness of alternative psychophysiological measures in differentiating psychopathy from general antisociality.

## Introduction

Severe antisocial behaviour can be observed across a wide span of disorders, including conduct disorder and antisocial personality disorder. Within the spectrum of antisocial disorders there is a group of individuals classified with psychopathy, which has traditionally been typified by disturbances in affective functioning combined with severe antisociality. In the past two decades, disturbed functioning in these two domains has been assessed with the Psychopathy Checklist-Revised (PCL-R; [1]), which has been the golden standard for the assessment of clinical psychopathy. The PCL-R measures behaviour reflecting interpersonal-affective functioning and antisociality and yields a total score indicating the presence of psychopathy. Studies assessing the cognitive counterparts of these behavioural indexes have linked psychopathy to impaired processing of affective information [2], and to disturbances in other non-affective cognitive domains such as learning [3] and attention [4]. In contrast, non-psychopathic antisocial behaviour has been linked to a broader range of problems in executive processing relative to psychopathy [29], [30]. The latter points out that while the concepts of psychopathy and generic antisociality show overlap on the behavioural level, they seem to differ in the cognitive processes that are affected and the extent to which these are deficient.

Attention is one of the cognitive processes that have been investigated extensively in comparative studies between psychopathy and non-psychopathy. There are numerous behavioural results indicating abnormalities in attentional processes that seem to be unique to PCL-R diagnosed psychopathy compared to non-psychopathic antisociality [5]. In contrast, relatively few studies have examined the electrophysiological correlates of attention in psychopathy [6]–[11]. A recent study using event-related potentials (ERPs) found that the abnormal allocation of attention in psychopathy seems to be due to disturbances at an early stage of selective attention, reflected by an increased positive ERP around 140 ms after stimulus presentation (P140; [11]). These ERP results were interpreted as additional support for the Response Modulation (RM) theory, which predicts that psychopathy is related to a tendency to over-allocate attention to goal-relevant information and to ignore potentially relevant secondary information. Apart from these early effects, selective attention is also involved in later stages of processing [12].

Previous ERP studies on attention in psychopathy have mainly focussed on this later aspect of attention by looking at components belonging to the P3-family [6]–[10], [13]. The term P3-family refers to a conglomeration of ERP components with a positive deflection occurring in a separate, much later time-window than the P140. The components belonging to the P3-family have been implicated in various functions such as attentional processing [14], inhibition [15] and error-processing [e.g. 16]. Two P3 potentials have been shown to be modulated by attentional allocation and task demands [17]. These components can be assessed using the oddball paradigm, in which infrequent target stimuli are presented in a string of frequent nontarget stimuli. Voluntary detection of the infrequent target stimuli elicits a P3 with a parietal distribution, also known as the P3b [18]. A variant of this task, the three-stimulus oddball paradigm, also includes the occurrence of highly salient task-irrelevant novel stimuli. In this version, participants respond to infrequent target stimuli but withhold their response to both infrequent novel and frequent standard stimuli. Task-irrelevant novel stimuli are known to elicit a P3 with a frontocentral distribution termed the P3a (or the novelty P3) [19]. The P3a reflects an involuntary automatic orienting of focused attention to novel stimuli and this mechanism is governed by anterior cingulate cortex (ACC) [20].

The results of the aforementioned studies on the P3 potentials in individuals with PCL-R diagnosed psychopathy have been inconclusive. Jutai et al. [6] investigated the P3b under single-task and dual-task conditions and did not find differences in amplitudes. In contrast, Raine and Venables [7] employed a continuous performance task and reported enhanced P3b amplitudes in subjects scoring high on psychopathy. Later studies by Kiehl et al. [8], [9] found the P3b to be reduced in psychopathic samples compared to non-psychopathic incarcerated offenders, as did Gao et al. [10] in a community sample of unsuccessful (caught) psychopaths. In sum, the P3b has been found to be reduced, normal and enhanced in samples scoring high on psychopathy.

Until now, only two studies specifically investigated the frontal P3 to novel oddballs in psychopathy [9], [10]. Kiehl et al. [9] reported the P3a to be reduced, but only in one of the two psychopathic samples tested and no differences were found in the other sample. Gao et al. [10] reported no differences in P3a amplitudes between controls, successful (uncaught) and unsuccessful psychopaths. Furthermore, a study on another frontal P3 component known as the NoGo P3 found reduced amplitudes in psychopathy [21], while a more recent investigation found the NoGo P3 to be unaffected in psychopathy [13]. Thus, the results on frontal components are also contradicting. One general explanation for these mixed results might be that the different tasks used tap into slightly different cognitive processes and these discrepancies are in turn reflected by differences in ERPs (for more details see [22]). In short, more research on the relationship between the P3s and PCL-R diagnosed psychopathy is needed in order to increase our understanding of these inconclusive results.

In sharp contrast to psychopathy, P3 findings in various *non-*psychopathic samples related to antisocial behaviour have shown much more convergence. In general, both the P3a and the P3b tend to be reduced in these populations, which include disorders such as substance abuse disorder [23], [24], conduct disorder [25], [26], and populations at risk of developing these types of disorders [27], [28]. A recent meta-analysis found a negative relationship between antisocial behaviour in general and the P3 [29]. It was suggested that the reduced P3 in antisocials reflects faulty utilization of neural resources, resulting in hampered processing of relevant information. However, it was pointed out that this deficiency might be less prominent in psychopathy. These results highlight the need to establish how well each of these two groups can recruit neural resources in order to process information that is relevant to the task at hand.

As processing of information is continuous and dynamic, one approach is to regard the P3 components as electrophysiological manifestations of neural recruitment during this process. More specifically, the automatic orienting of focussed attention reflected by the P3a facilitates the allocation of attentional resources to successive memory storage operations in the hippocampal formation. The output is then passed on to the parietal cortex. This latter, controlled attentional process in parietal regions is reflected by the P3b [20]. This interactive mechanism between frontocentral and parietal areas is indicative that monitoring events is a continuous process. Although the distributions are frontocentral for the P3a and parietal for the P3b, an electrophysiological response to targets can also be observed in frontocentral areas, albeit smaller in amplitude relative to novels. The opposite pattern can be observed in parietal areas. More specifically, the P3 to novels is larger than the electrophysiological response to targets in frontocentral areas, while the P3 to targets is larger than the response to novels in parietal areas. To our knowledge, this dynamic switch in electrophysiological pattern resulting from the interplay between frontocentral and parietal areas has not been explicitly assessed before in either healthy or patient samples. Examining whether the switch in pattern is present in the ERPs to targets and novels in frontocentral in relation to parietal regions could yield valuable information about the quality of neuronal recruitment and the extent to which the cognitive processing driving these potentials are functionally affected. Thus, the current approach offers a more sensitive electrophysiological measure for examining and comparing the quality of cognitive processing in psychopathic and non-psychopathic clinical samples.

The main goal of the present study was to assess cognitive processing of rare novel and target events in psychopathy relative to a non-psychopathic sample of institutionalized offenders and a group of matched healthy control individuals. Based on the converging findings in non-psychopathic samples, a diminished P3a to novel stimuli was expected in non-psychopathic offenders compared to both psychopathic and healthy individuals. In contrast, due to the lack of group differences in the majority of the samples in which a frontocentral P3 was assessed in clinical psychopathy [9], [10], [13], combined with reports on intact automatic processing in ACC [31], the P3a was expected to be intact in psychopathic subjects relative to the non-psychopathic participants (thus similar to healthy controls). Second, reductions were found in three out of five reports on the P3b in psychopathy and in a large amount of studies in non-psychopathic samples of antisocials, and we subsequently predicted reduced P3b amplitudes in both non-psychopathic and psychopathic offenders relative to healthy controls. Finally, the quality of processing and attentional allocation during the continuous monitoring of infrequent stimuli was also investigated in the offender groups by examining the switch in the pattern of the ERPs to targets and novels in frontocentral and parietal areas.

## Methods

### Participants and procedure

Two offender groups were recruited from the population of the Pompestichting Forensic Psychiatric Institute Nijmegen, The Netherlands. The Pompestichting is a clinic for individuals who have committed serious criminal offences in connection with having a DSM-IV axis-I and/or axis–II disorder. Placement in such clinics falls under a measure known as ‘Ter Beschikking Stelling’ (TBS). TBS is a treatment measure on behalf of the state and is not a punishment, but an entrustment act for offenders with mental disorders. The TBS measure is ordered by the court and offers an alternative to confinement in psychiatric hospital or long-term imprisonment, with the aim of balancing treatment, security and protection.

The offenders were selected based on prior history and information about their clinical status. Twenty offenders diagnosed with psychopathy and twenty-three non-psychopathic offenders were included in this study. Psychopathy was assessed with the PCL-R, which consists of twenty items representing different behavioural characteristics that are scored as being absent (0), moderately present (1) or clearly present (2) based on file information and a semi-structured interview [32]. The PCL-R was administered by trained psychologists upon admittance to the Dutch forensic mental health system. Therefore, available PCL-R scores were retrieved from participants' files. In Europe, a cut-off score of 26 is usually maintained for the PCL-R [e.g. 31,33; but see 34], thus offenders with a PCL-R score ≥26 were included in the psychopathic group and those with a score <26 in the non-psychopathic patient group (Table 1).

**Table 1:** Group characteristics for the psychopathic, non-psychopathic and the control group.

Characteristic	Psychopathy (n = 20)	Non-psychopathy (n = 23)	Healthy controls (n = 16)
Age	40 (10)	37 (8.8)	37 (6.7)
Educational Level	2.5 (0.6)	2.6 (0.6)	2.9 (0.4)
PCL-R Score	30 (4.2)*	15.7 (4.8)	

Group means are reported with their standard deviation between brackets. Significant Group differences are flagged.

Sixteen healthy control participants were recruited through advertisements. The control group consisted of volunteers without criminal records and a history of psychiatric disorders. Because none of our healthy controls had criminal records, which are essential for reliably assessing PCL-R scores, the PCL-R scores were not assessed in the healthy control group. All participants were males and the groups were matched for age and educational level. Educational level was categorized into three subdivisions based on the Dutch educational system (level 1 =  primary education, level 2 =  secondary education, level 3 =  higher education) [31].

All subjects participated in two sessions; a screening session and a test session. During the screening session, compliance to the inclusion criteria was determined *for all three groups* using the Dutch version of MINI Psychiatric Interview [35] and the SCID-II [36]. In addition, information from criminal records was used for the offender groups. Participants were excluded if one or more of the following disorders were present: depressive disorder, bipolar disorder, schizophrenia, schizoaffective disorder, schizophreniform disorder, delusional and other psychotic disorders, schizoid or schizotypical personality disorder, attention deficit hyperactivity disorder, antisocial personality disorder and/or psychopathy were excluded only in healthy volunteers, and first degree relatives with DSM-IV axis I schizophrenia or schizophreniform disorder. Other exclusion criteria were the use of intoxicating substances or psychotropic medication within the week preceding the experimental session, and a positive result on any of the unannounced urinal drug tests that were randomly administered. All assessments were conducted by trained psychologists. If the criteria were met, an appointment was made with the participants for the test session in which behavioural and EEG data were acquired.

### Ethics statement

All participants received written information about the experiment, a financial compensation, and gave written informed consent. Potential participants were allowed a period of at least two weeks to consider and discuss their participation before signing the following consent form: *By signing this form I confirm that I voluntarily give consent to participate in this study. I have received and read a copy of the information for participants. I am informed about the study and have had enough time to think about my participation. My questions have been answered satisfactorily. I am aware that I can withdraw my consent at any time without giving any reason and without any adverse consequences on my further treatment.* For each participant, the experimenter signed the following section: *I confirm that this participant has been given explanations concerning the nature, purpose and possible risks of this research, and has voluntarily agreed to participate in the study. The participant confirmed his voluntary consent by signing above.*

For each potential participant from the offender population, the full capacity to consent was established by consulting the head therapist in charge of the participant's treatment and care. Potential participants lacking the capacity to consent themselves (i.e. having a low level of competence) as indicated by the presence of mental retardation or any psychiatric condition associated with reduced competence, or not meeting the inclusion criteria were still eligible for treatment. Thus, the decision to participate did not affect the patient's treatment or care in any way. The protocol was approved by the local medical ethical committee (Commissie Mensgebonden Onderzoek Regio Arnhem-Nijmegen) and the rights of the participants were protected.

### Task and Design

A three-stimulus oddball paradigm was employed in order to investigate both variants of the P3. Subjects were seated at approximately 75 cm from a 100 Hz monitor and the stimuli were presented in the centre of the display in black against a white background. The stimuli consisted of either the letter ‘S’, the letter ‘H’ or one of 40 different non-letter ASCII characters with font size 24 and font type Arial. Participants were instructed to use their right index finger to press a designated button on a button box whenever the letter ‘S’ (Target, 10%) appeared and to withhold responses if the stimulus was either an ‘H’ (Standard, 80%) or another unique character (Novel, 10%). Participants were not informed about the occurrence of rare novel stimuli in the task. Four hundred trials were presented, divided in 4 blocks of 100 trials. Stimuli were presented for 250 msec and followed by a 1500 msec response window before the next stimulus was presented.

### Apparatus and recordings

Electrophysiological data were collected using 27 active electrodes (ActiCap, Brain Products, Munich, Germany) arranged according to a variation of the 10–20 system. Abralyt 2000 abrasive gel (EasyCap, Herrsching, Germany) was used for the conduction of signals to the electrodes. Vertical eye movements were recorded by placing electrodes above and below the left eye and horizontal eye movements were registered at the outer canthi of the eyes. Electrophysiological data was acquired at 500 Hz without filtering with the QuickAmp amplifier (Brain Products) and the electrodes were referenced to the left ear during signal acquisition.

### EEG data processing

ERP data were filtered offline using a .02–20 Hz filter and re-referenced to the average of the linked ears. EOG artefacts were removed using Independent Component Analysis [46]. Additional artefact rejection scans were conducted in order to detect other types of artefacts remaining in the data. Amplitudes exceeding ± 50 µV were labelled as artefacts and removed from the dataset and a minimum of 15 artefact-free trials for each participant in each condition was set as a condition for inclusion [37], but artefact rejection yielded an average of 36 novel and 38 target trials per participant. Subsequently, activity associated with each type of stimulus was averaged separately in epochs starting 200 msec prior to stimulus presentation and ending 700 msec after stimulus onset. Segments were baseline corrected to a 200 msec pre-stimulus interval.

The P3s were detected with automatic algorithms at electrode sites FCz and Pz. As the P3a has been reported both at Fz [23] and at FCz [38] in these types of populations, we first explored which of these two frontal electrodes showed larger amplitudes. These were larger at FCz. The most positive peak between 275–575 msec following stimulus-onset was determined for the P3a [24] and between 300–700 msec for the P3b. The responses to the frequently occurring standard stimuli were not included in the analyses because detailed inspection of the data indicated that not all participants had a pronounced electrophysiological reaction to this type of stimulus. Therefore, it was not possible to execute peak detection for the standard stimuli which would yield reliable results for each individual.

**Table 2:** Behavioural results for the psychopathic, non-psychopathic and the control group.

	Psychopathy (n = 20)	Non-psychopathy (n = 23)	Healthy controls (n = 16)
Reaction time	470 (76)	479 (70)	398 (54)*
Correct hits	39.4 (1)	39.7 (.6)	39.7 (.7)
False alarms	0.5 (0.7)	0.17 (0.4)	0.38 (0.8)
Errors to non-targets	0.65 (.8)	0.22 (.4)	0.44 (.8)

Group means are reported with their standard deviation between brackets. Reaction times are reported in msec and accuracy measures in counts. Significant group differences are flagged.

**Figure 1:**
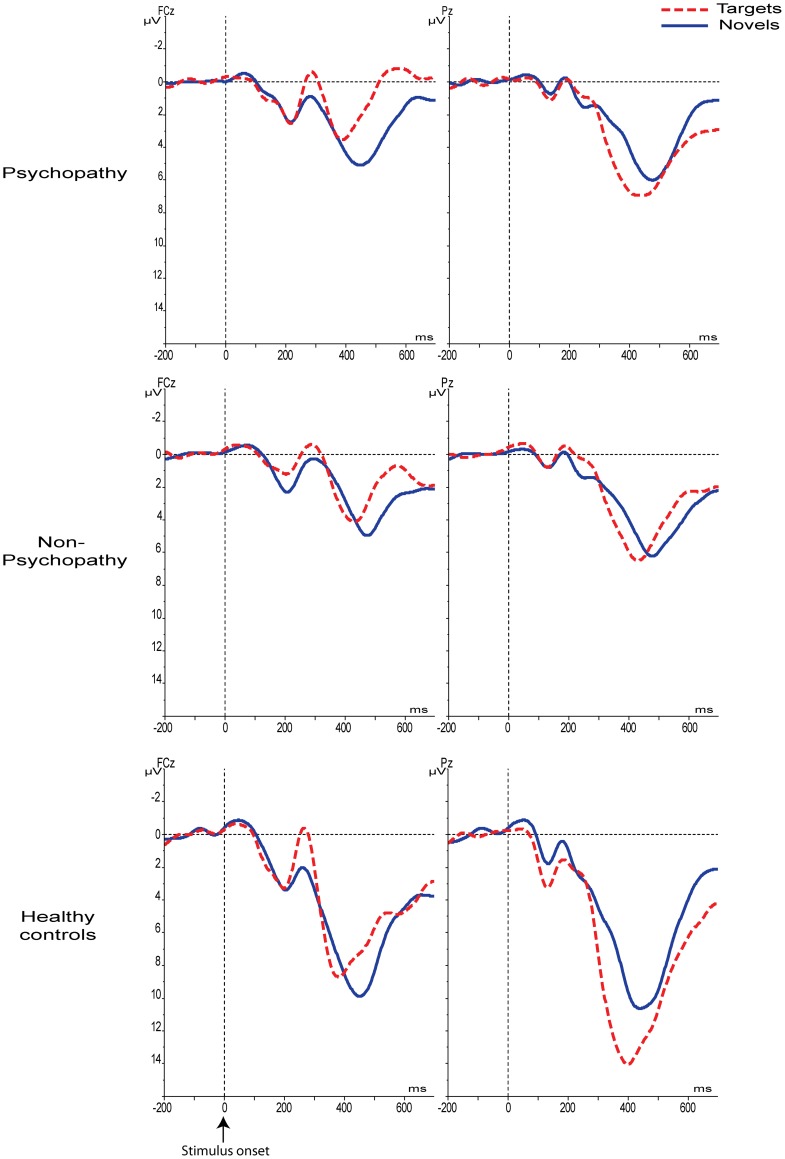
Grand average stimulus-locked waveforms for the P3a at FCz and the P3b at Pz for each group separately.

### Statistical analyses

For ERP analyses, the individual mean amplitudes were entered in a repeated measures General Linear Model (GLM) with Stimulus Type (Novel, Target) and Location (FCz, Pz) as within-subject factors and Group (Controls, Non-psychopaths, Psychopaths) as between-subjects factor. Behavioural data were investigated by entering reaction times (RTs) to targets in a univariate GLM with Group as between-subject factor. Accuracy data were divided in correct responses to targets, incorrect button presses to novels (false alarms), and errors to non-targets (commission errors) and analysed with Kruskal-Wallis tests because the data were not normally distributed.

**Figure 2:**
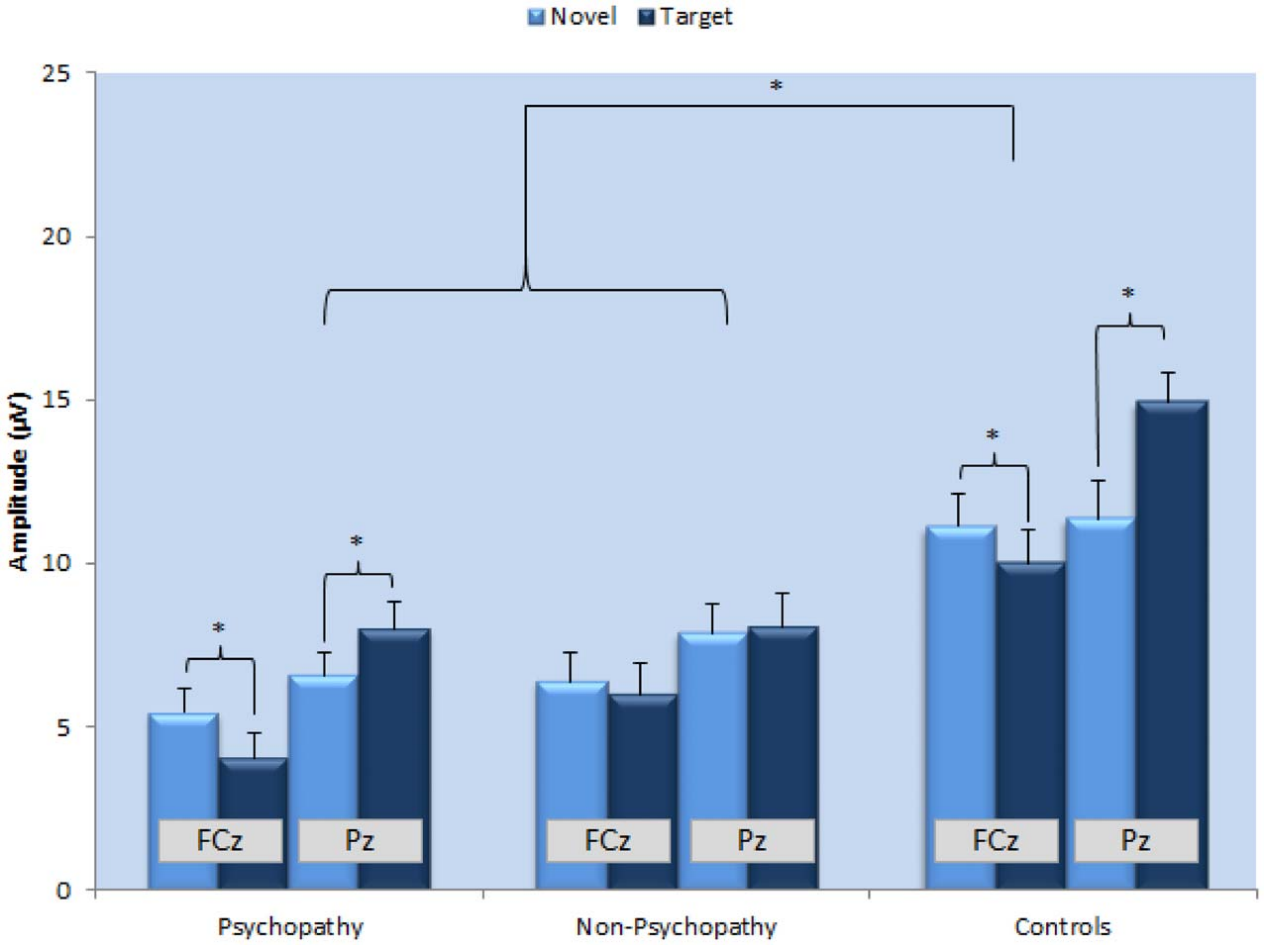
Average peak amplitudes for novels and targets at FCz and Pz for the psychopathic, non-psychopathic and control group, respectively.

## Results

### Behavioural results

RT analyses revealed a main effect for Group [*F*(2, 56) = 7.32, *p* = .001]. Healthy controls showed shorter RTs (399 msec; all *p*'s<01; Table 2) than the psychopathic group (470 msec) and the non-psychopathic group (479 msec), while the two patient groups did not differ (*p* = 902). The groups did not show any differences in amount of correct hits [ χ^2^ (2, *N = *59)  = .558, *p* = .757], false alarms [χ^2^ (2, *N = *59)  = 3.04, *p* = .218], or in the total number of responses to non-targets [χ^2^ (2, *N = *59)  = .421, *p* = 122].

### ERP results

Initial analyses showed that there was a main effect for Location [*F*(1,56) = 15.6, *p*<001] indicating higher overall amplitudes at Pz (9.4 µV, SD = 5.0) compared to FCz (7.4 µV, SD = 4.8). There was no main effect for Type [*F*(1,56) = .422, *p* = .519]. As expected, there was a significant interaction for Location×Type [*F*(1,56) = 47.2, *p*<001], indicating that the mean P3 amplitude to novels (7.9 µV, SD = 5.0) was larger at FCz compared to targets (6.9 µV, SD = 5.1; *t*(58) = 2.59, *p* = .012), while amplitudes to targets were maximal at Pz (10.0 µV, SD = 5.2) compared to novels (8.7 µV, SD = 4.2; *t*(58) = −3.1, *p* = .003). The main effect for Group revealed smaller overall amplitudes in the offender samples [*F*(2,56) = 11.1, *p*<001; Figure 1]. Importantly however, the Location×Type×Group interaction also reached significance [*F*(2,56) = 9.79, *p*<001].

To identify the source of the latter significant 3-way interaction, separate GLMs were carried out for each group, with Type and Location as within-subject factors. The results revealed significant Location×Type interactions for both the psychopathic and the control group (all *F's*>13.3, all *p's*<01). Further examination of this two-way interaction revealed that also within these two groups, peaks to novels were significantly larger than targets at FCz, while targets elicited significantly larger amplitudes than novels at Pz (one-sided paired sample t-tests: all *p's*<05; see Figure 2). In contrast, the Location×Type interaction was not significant for the non-psychopathic offenders, [*F*(1,22) = 1.31, *p* = .265], indicating that the non-psychopathic group did not differentiate between novels and targets at FCz nor at Pz (see Figures 1 and 2).

## Discussion

The aims of the present study was to investigate and compare the P3 to novel events (P3a) and the P3 to infrequent targets (P3b) between groups of offenders with and without psychopathy and healthy controls, and to compare the groups on the ability to differentially allocate (late) attention and process various stimulus types at an electrophysiological level. The results show that both psychopathic and non-psychopathic offenders generally exhibit reduced P3a and P3b amplitudes compared to healthy individuals, but do not differ from each other in overall amplitudes. The findings in the non-psychopathic offenders corroborate previous reports of reduced P3s in general (non-psychopathic) antisociality [29]. At first glance, the results on the P3a in the group with psychopathy would seem in contrast to our hypothesis that the amplitude of the P3a should be similar to that of the healthy controls and would also be consistent with previous outcomes showing P3a reductions in psychopathy [9]. Importantly however, the present findings suggest that a more subtle difference exists between the offender groups that is not captured by traditional methods assessing overall peak estimates. In spite of the overall reduction in P3 amplitudes, the psychopathic group showed a larger P3 to novel relative to target stimuli in frontocentral areas and larger P3 amplitude to targets compared to novels in parietal areas, thus resembling the healthy individuals on this aspect. These findings indicate that psychopathic individuals are capable of monitoring and allocating late selective attention accordingly to various types of infrequent stimuli, even in the light of an overall reduction in deployment of attentional resources. The latter seems not to be the case in the non-psychopathic group of offenders.

The ability to still differentiate novels and targets found in the group with psychopathy is consistent with the claim that psychopathy is related to enhanced processing capabilities [29]. It is plausible that they were showing superior processing capabilities, because their level of processing ultimately leads to the same psychophysiological pattern as healthy controls, while deploying fewer resources. This idea converges with previous claims that the monotonous nature of the oddball task might not be stimulating enough to fully trigger the attentional resources of psychopathic individuals and could also be an explanation for the lack of differences between the two offender groups on overall P3 amplitudes. Future studies using more complex paradigms combined with more fine-grained stimulus-level ERP analyses could shed more light on this issue.

It is also worth considering our results in light of the attention-based RM hypothesis. The traditional formulation of this hypothesis postulates that the abnormal behaviour seen in psychopathy is due to abnormalities in the automatic allocation of attention to secondary but meaningful information to current goal-directed behaviour [4]. Thus, psychopathic individuals fail to attend to secondary information that competes for the occupation of the focus of attention with information that is central to current goal-directed behaviour. Based on this general definition it could initially be predicted that psychopathy should be related to reduced attentional allocation to non-relevant novel events and a tendency to overfocus on the target stimuli in our task, which should be reflected by reduced P3s to novelty relative to the P3s to targets in both frontocentral and parietal areas. Our findings do not seem to support this prediction as the group with psychopathy did not show larger ERPs to targets at both locations. One explanation could be that our task was not suitable to test the mechanisms that have been claimed to be related to the deficient response modulation in psychopathy. Stimuli were presented in succession, which means that there was no competition between peripheral and central information for occupying the focus of attention. Furthermore, recent work within this framework has narrowed down the abnormalities in allocation of attention in psychopathy to an early attentional bottleneck that occurs in a much earlier time-window relative to the P3 [11], [39]. Baskin-Sommers et al. [11] found psychopathic inmates to show larger ERP amplitudes implicated in early attentional processing, suggesting superior allocation of attention in this early stage. It is possible that this superiority caused an increased deployment of cognitive resources in an early stage of processing in order to differentiate between the stimuli in the group with psychopathy, reducing the need for engaging cognitive resources for differentiation later stages in the timeframe of the P3. Thus, the presence of an anomalous early attention bottleneck as postulated by recent specification of the RM hypothesis could also explain our findings showing intact stimulus differentiation in spite of reduced overall amplitudes in the group with psychopathy.

In contrast to the psychopathic group, non-psychopathic subjects failed to show appropriate type-dependent modulation of attention and seemed to disengage their resources during processing, which was especially evident in the total lack of differentiation in parietal areas (Figure 2). These results are in line with previous evidence linking impairments in cognitive processing and the P3 to non-psychopathic antisociality [29]. Also, one tentative hypothesis is that this deficiency in disentangling information might be related to greater perceived ambiguity in the interpretation of information, which in turn may result in hostile and inappropriate behaviour often seen in these types of (non-psychopathic) populations [e.g. 40]. Future studies specifically designed to address this matter should explore this possibility.

The results also support the notion that although offenders with and without psychopathy clearly show overlap in covert behaviour and psychopathology, they may still differ on other aspects (such as the extent to which specific personality traits are present) and in their neurocognitive make-up [cf. 41]. The combination of our electrophysiological and our behavioural results add support to this claim. The behavioural findings point out that the healthy control group showed shorter RTs compared to the offender samples, while the offender groups did not differ from each other on any behavioural measure. Also, all groups showed very high levels of accuracy and did not differ on any of these measures. This pattern of performance could be accounted for in terms of a speed/accuracy trade-off, which required the offenders to slow down in order to achieve normal accuracy that is comparable to that of the healthy controls. This interpretation would be consisted with previous reports of poor behavioural performance in both non-psychopathic antisociality and psychopathy [30], [42]. However, the group difference in the discrimination of novels and targets reflected by the ERPs indicates group dissimilarities in the neurocognitive processing preceding the observed behaviour. In a recent investigation of the interplay between inhibitory control and affective processing in psychopathy and non-psychopathy it was also found that both groups showed comparable behavioural performance while ERPs showed significant group differences in cognitive performance [43]. The absence of group differences in behaviour might be due to the simplicity of the tasks used both in the present study and that by Verona et al. [43]. All together, these results converge with previous claims that these groups form two related but separable populations within the spectrum of antisocial personality disorders [9], [41], with non-psychopathic antisociality being more prone to deficient cognitive processing in general relative to psychopathy [29].

One potential limitation is that it could be argued that the size of our samples might have led to insufficient statistical power. However, our samples were large enough to detect between-group effects, within-group effects and the interactions of interest with high levels of significance in our GLMs. Another potential limitation comes from the argument that the diminished cognitive processing (reflected in this case by the reduced P3s) found in the offender groups are related to a more general reduction in cognitive well-being during incarceration [44], [45]. As countries differ in their penitentiary regimes, in some countries inmates regularly remain confined to their cells for the great majority of the day or are deprived in other ways. This could debatably lead to less exercising of their cognitive skills. In our case, we believe that it is unlikely that incarceration itself is responsible for our results. The Dutch forensic psychiatric system is unique in that it mimics everyday life outside the forensic clinics, requiring patients to work, participate in therapies, study, exercise, etc., throughout the day. Moreover, some of the offenders were in the resocialization trajectory, meaning that they were working outside the clinic and participated in society on a daily basis while still under surveillance and care of the institute. Therefore, we do not believe that the differences found relative to our healthy control group can be purely attributed to incarceration.

### Conclusion

In sum, this study directly compared the P3a and P3b in healthy subjects, non-psychopathic offenders and psychopathic individuals. The findings show that both psychopathic and non-psychopathic offenders exhibit reduced P3 amplitudes to rare events in both frontocentral (P3a) and parietal areas (P3b) relative to matched healthy controls. This is generally indicative of a reduced ability to allocate late selective attentional resources to infrequent events. Importantly however, the current study provides evidence for a dissociation between the two offender groups on a more detailed level. While the psychopathic group did show normal differentiation in attentional allocation to infrequent task-relevant and task-irrelevant stimuli, the non-psychopathic sample did not show this pattern. These results also highlight the advantage and importance of assessing electrophysiological processes on a more detailed level when comparing populations known to show deficiencies reflected in specific ERP components. Comparing groups based on grand average ERPs (calculated across all subjects within a specific sample) is very useful in ascertaining whether a specific group shows larger or smaller ERP amplitudes. However, this method conveys less information about the health of the cognitive mechanisms that drive the individual ERPs. Future studies employing alternative approaches to data analyses would help disentangle the neurocognitive underpinnings of different psychiatric populations collectively marked as antisocial, in order to increase our understanding of this heterogeneous and relatively opaque class of personality disorders.
